# Hydrogen-accelerated spontaneous microcracking in high-strength aluminium alloys

**DOI:** 10.1038/s41598-020-58834-6

**Published:** 2020-04-06

**Authors:** Tomohito Tsuru, Kazuyuki Shimizu, Masatake Yamaguchi, Mitsuhiro Itakura, Kenichi Ebihara, Artenis Bendo, Kenji Matsuda, Hiroyuki Toda

**Affiliations:** 1 Nuclear Science and Engineering Center, Japan Atomic Energy Agency, Tokai-mura, Ibaraki, 319-1195 Japan; 2 Elements Strategy Initiative for Structural Materials, Kyoto University, Sakyo-ku, Kyoto, 606-8501 Japan; 3 PRESTO, Japan Science and Technology Agency, Kawaguchi, Saitama, 332-0012 Japan; 4 Department of Mechanical Engineering, Kyushu University, Fukuoka, Fukuoka, 819-0395 Japan; 5 Center for Computational Science and e-Systems, Japan Atomic Energy Agency, Tokai-mura, Ibaraki, 319-1195 Japan; 6 Graduate School of Science and Engineering for Research, University of Toyama, Toyama, Toyama, 930-8555 Japan

**Keywords:** Metals and alloys, Atomistic models

## Abstract

Aluminium alloys are re-evaluated as most feasible way to satisfy the industrial needs of light-weight structural materials. However, unlike conventional structural metals such as iron and titanium, aluminium does not have easily accessible secondary phases, which means that aluminium-based alloys cannot be strengthened by harnessing multiple phases. This leaves age hardening as the only feasible strengthening approach. Highly concentrated precipitates generated by age hardening generally play a dominant role in shaping the mechanical properties of aluminium alloys. In such precipitates, it is commonly believed that the coherent interface between the matrix and precipitate does not contribute to crack initiation and embrittlement. Here, we show that this is not the case. We report an unexpected spontaneous fracture process associated with hydrogen embrittlement. The origin of this quasi-cleavage fracture involves hydrogen partitioning, which we comprehensively investigate through experiment, theory and first-principles calculations. Despite completely coherent interface, we show that the aluminium–precipitate interface is a more preferable trap site than void, dislocation and grain boundary. The cohesivity of the interface deteriorates significantly with increasing occupancy, while hydrogen atoms are stably trapped up to an extremely high occupancy over the possible trap site. Our insights indicate that controlling the hydrogen distribution plays a key role to design further high-strength and high-toughness aluminium alloys.

## Introduction

The design of strong, lightweight structural materials is important for energy saving and sustainability. Although aluminium (Al) alloys are prime candidates to meet the increasing demand for lightweight materials for use in commercial applications, the fundamental approach to increasing the strength and functionality of these alloys has not been improved since age hardening of duralumin was first developed by A. Wilm in 1906^1^. The strength of Al alloys generally depends on the distribution of fine precipitates nucleated from a supersaturated solid solution in the Al matrix. Traditionally, Al-alloy strength is increased by adding major alloying elements (Si, Mg, Zn, Cu) and some minor elements (Ni, Si) that nucleate precipitates. The trade-off relationship between strength and susceptibility to hydrogen embrittlement (HE) is also recognized as a common problem with metals and high-strength Al alloys^2–5^. The hydrogen environment behaviour is still unknown because hydrogen is not likely to be soluble or to nucleate hydrides in an Al matrix as compared with other lightweight materials such as titanium^6^.

The 7xxx series of alloys are high-strength Al alloys that contain zinc (Zn) and magnesium (Mg) as the major alloying elements for age hardening as well as a small quantity (below 1 wt%) of other elements for corrosion resistance^7^. In these alloys, MgZn_2_ precipitates are formed according to the following transformation through the ageing process: supersaturated solid solutions (SSSS) =  > GP zone =  > η’ =  > η-MgZn_2_^8,9^. Researchers have been working on a number of efforts to reduce the hydrogen concentration as a way to prevent HE^10^, as the presence of a supersaturated hydrogen concentration in practical use generally enhances ductile fracture due to the high-density micropore distribution. More recently, however, a small portion of dissolved hydrogen has been found to cause an anomalous brittle fracture known as “quasi-cleavage” fracture^11,12^.

In case of martensitic steel in which quasi-cleavage fracture was often observed, this type of cleavage fracture is caused by hydrogen-enhanced and plasticity-mediated decohesion at the interface^13–15^. In Al alloys, in contrast, this quasi-cleavage fracture exhibits distinctive features. The fracture behaviour cannot be explained by any other fracture mechanism in that the crack propagation occurs along the (111) plane, corresponding to the slip plane, although the plastic deformation does not mediate the crack propagation. In addition, the quasi-cleavage fracture observed in Al alloys differs from that of well-known fracture modes in metals such as hydrogen-induced cracking^16^, which is generally caused by hydrogen-enhanced decohesion (HEDE) at cleavage planes and grain boundaries^17–20^, hydrogen-induced local plasticity (HELP)^21–23^, and hydrogen-mediated micropore distribution^10,24,25^.

Here we report on the HE mechanism related to quasi-cleavage fracture unique to Al alloys using three-dimensional observation technique and atomistic simulations. Synchrotron X-ray imaging and scanning electron microscopy (SEM) with energy dispersive X-ray spectroscopy (EDS) analysis are used to capture the characteristic features of the fracture and, in particular, to elucidate the relationship between the fracture surface and coherent surface planes of the η-phase precipitates. We focus on the hydrogen embrittlement of high-strength Al-Zn-Mg alloys and estimate the equilibrium partitioning of hydrogen in this Al alloys using a thermodynamic model based on first-principles density functional theory (DFT) calculations.

## Results and Discussion

### Experimental observation of brittle fracture mode

**Figure 1:**
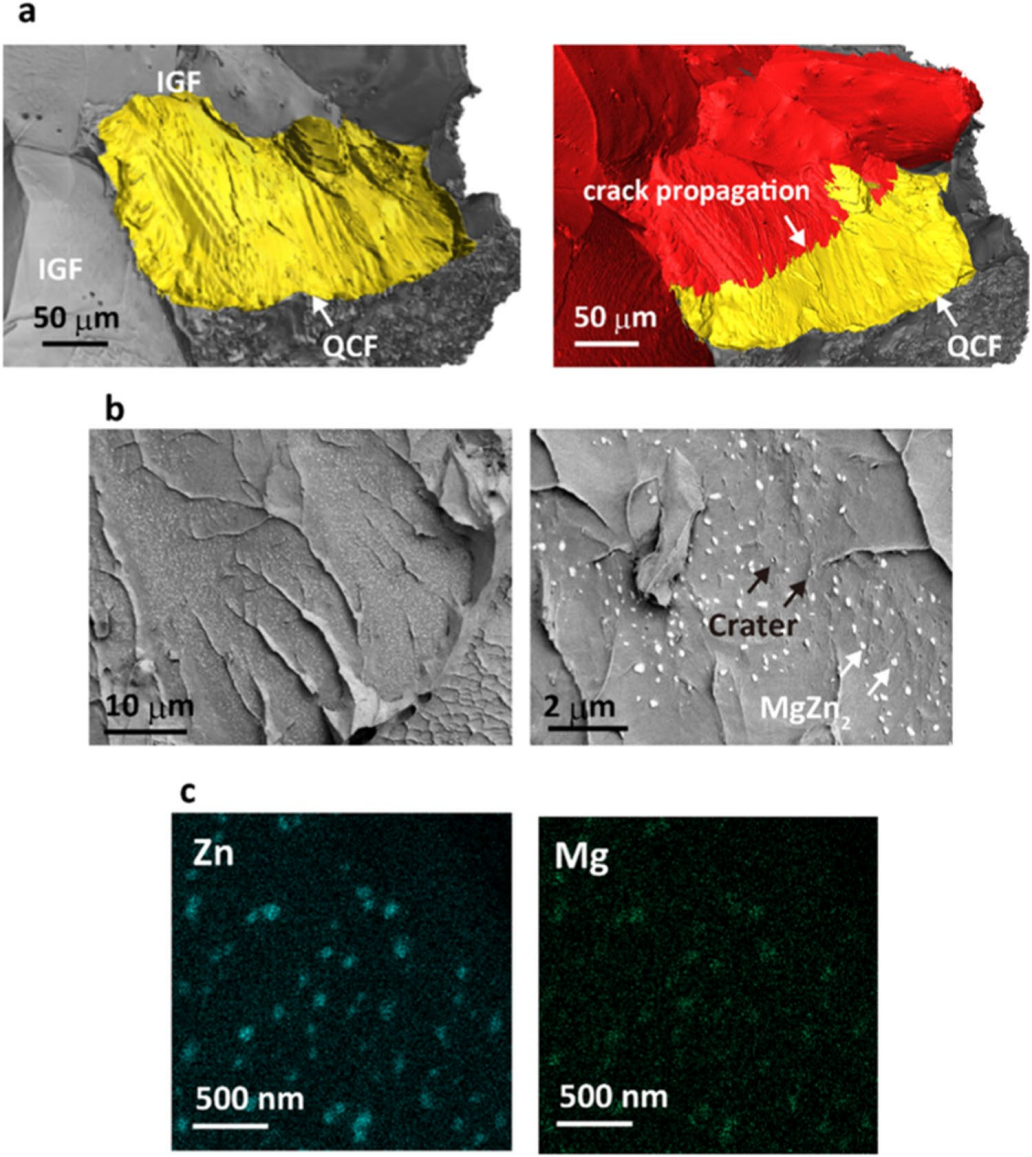
Experimental observation of the fracture surface of an Al-Zn-Mg alloy. (**a**) Three-dimensional tomographic images of fractured Al-Zn-Mg alloy samples captured by synchrotron X-ray tomography. Two fracture modes – intergranular fracture (IGF) and quasi-cleavage fracture (QCF) – can be observed on the fracture surface. Crack propagation can be also captured by tomographic image on the right, where the image was obrained at *ε* = 11% (near *τ*_UTS_). The crack is found to propagate gradually and undulatory along various quasi-cleavage facets at which hydrogen atoms are sufficiently trapped. (**b**) Low-voltage SEM images of this fracture surface, in which very dense white and black dots correspond to an MgZn_2_ precipitate and the crater of the precipitate, respectively. (**c**) EDS mapping of Zn and Mg on the fracture surface, indicating a clear region of precipitate.

Al-10.0Zn-1.2Mg alloys were designed to investigate fracture behaviour in a hydrogen environment. In the present study, we investigated brittle fracture using following specimen. We prepared tensile specimens containing a variety of defects (vacancy, dislocation, grain boundary and precipitate) in which hydrogen diffuses to the trap sites (see the methods section for details). Figure 1 summarises the observations of the fracture surface obtained in the tensile test. The fracture surface obtained by synchrotron X-ray tomography, as shown in Fig. 1(a), clearly corresponds to brittle fracture. According to the tomography images, the crack is found to propagate gradually and undulatory along various quasi-cleavage facets at which hydrogen atoms are sufficiently trapped. Interestingly, two fracture modes were also observed in the fracture surface – intergranular fracture (IGF) and quasi-cleavage fracture (QCF) – where crack propagation tends to proceed selectively depending on the tensile direction and local stress condition around the crack tip. IGF is caused by grain boundary decohesion due to hydrogen segregation^26,27^.

The fracture surface of the QCF has no-dimple and no-planar morphologies and is unique because the observed fracture surface cannot be explained by any other fracture modes. With regard to the QCFs, low-voltage SEM images on this surface are shown in Fig. 1(b). Surprisingly, very dense white particle are observed on the fracture surface. The particle was identified as MgZn_2_ precipitate through EDS mapping, as shown in Fig. 1(c). The enlarged view of the fracture surface shown in Fig. 1(b) indicates that the fracture occurs along the Al–MgZn_2_ precipitate interface given that almost the same number of craters as precipitates were observed on the fracture surface. The typical image of Al–MgZn_2_ interface are preliminarily investigated by transmission electron microscopy (See Supplementary Information). This tells us that the location of craters in one fracture surface correspond to that of precipitates in the other fracture surface. These experimental observations suggest that hydrogen trapping at the Al–MgZn_2_ precipitate interface induces decohesion and originate quasi-cleavage crack, promoting HE behaviour. Whether or not hydrogen atoms preferably segregate into the Al–MgZn_2_ precipitate interface depends on the magnitude correlation of the binding energy between hydrogen and each defect structure. We thus evaluated the tendency of hydrogen trapping at various sites using first-principles calculations.

### Binding energy of hydrogen at various trap sites

It is well known that the Al–MgZn_2_ interface, which plays enhancing the strength of alloys, does not influence the degradation of fracture behaviour in terms of interfacial decohesion because the η-phase MgZn_2_ precipitate nucleates the completely coherent interface with the Al (111) plane. This kind of interface generally does not tend to absorb interstitial atoms since the coherent interface contributes little to the increase in free volume. However, the fracture surface of the QCF mode indicates decohesion at the Al–MgZn_2_ interface as a result of hydrogen segregation.

**Figure 2:**
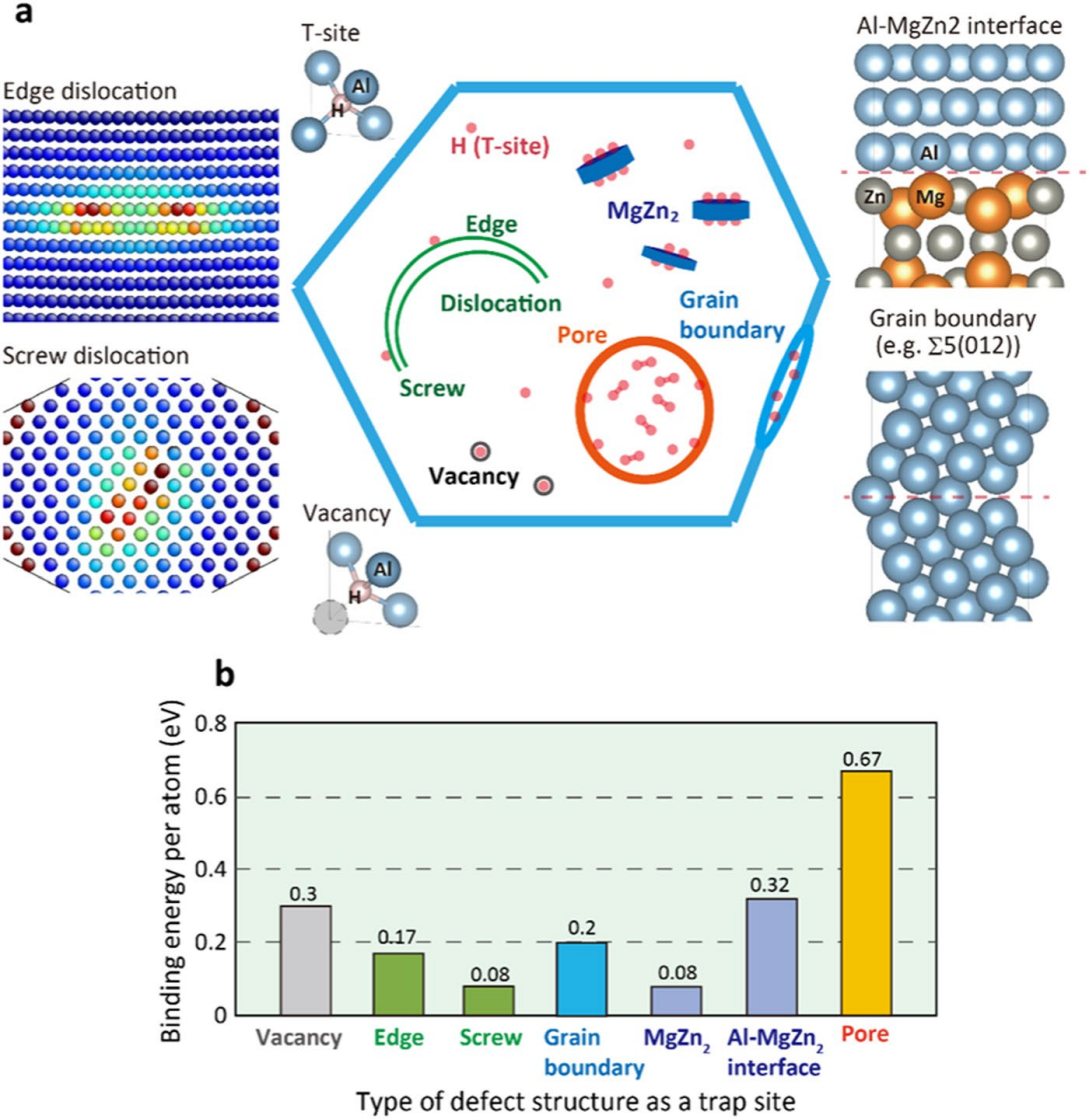
Schematic image of possible trap sites of hydrogen and their binding energy. (**a**) Schematic showing various defect structures as trap sites of hydrogen in Al alloys. Atomic models of these defect structures used for DFT calculations, where T-site corresponds tetragonal site. (**b**) Binding energy per atom between hydrogen and defect structures such as vacancy, edge/screw dislocations, grain boundary, and η-MgZn_2_ precipitate evaluated by first-principles calculations.

We comprehensively investigated the binding energy between hydrogen and various defect structures such as vacancy, edge/screw dislocations, grain boundary, and η-MgZn_2_ precipitate. The results are shown in Fig. 2(a). Atomic models of defect structures for DFT calculations at which hydrogen atoms are to be trapped are given in the same figure. The computational setup for the DFT calculations is described in the methods section. The most stable interface is expected to be reached by minimizing the elastic strain energy associated with the difference in the lattice constants between the Al matrix and MgZn_2_, and is found to be the η2 interface, which is consistent with the high-resolution transmission electron microscopy (HRTEM) observations^28,29^.

As a result, the η2 interface was taken as the target interface, where the η2 interface has a coincidence axis along ${\rm{A}}{\rm{l}}[1\bar{1}0]//{{\rm{M}}{\rm{g}}{\rm{Z}}{\rm{n}}}_{2}[\bar{1}100]$ and ${\rm{A}}{\rm{l}}{\rm{A}}{\rm{l}}[\bar{1}12]//{{\rm{M}}{\rm{g}}{\rm{Z}}{\rm{n}}}_{2}[11\bar{2}0]$ normal to the interface (Al(111)//MgZn_2_ (0001)^8^. The binding energies with all trap sites are summarized in Fig. 2(b), where the binding energies are defined as the difference in the energy between defects and stable tetrahedral sites in bulk Al. According to our calculations, hydrogen atoms are definitely trapped at the grain boundary through internal diffusion^30^. Both edge and screw dislocations do not have a significant influence on hydrogen trapping, while hydrogen changes the edge/screw character of dislocations and slip planarity^31–33^. On the other hand, the binding energy of the inner region of the MgZn_2_ crystal is extremely small (the biding energy at most stable site is 0.079 eV), which indicates that hydrogen tends not to be absorbed into the precipitate due to the unstable sites in MgZn_2_^34^. Interestingly, the results show that the binding energy at the Al–MgZn_2_ interface is higher than that of the grain boundary and vacancy^30,34^, even if the interface remains completely coherent (no lattice mismatch) and the free volume around the interface is small. The Al–MgZn_2_ interface is therefore one of the most favourable trap sites among possible defect structures in Al alloys such as vacancy, edge/screw dislocation and grain boundaries. Incidentally, the significant stability of the pore region, including of the surface and H_2_ molecule, implies that hydrogen segregation tends to occur spontaneously, stabilizing the surface and forming H_2_ molecules in spite of the highly concentrated hydrogen around defects.

### Hydrogen partitioning

**Figure 3:**
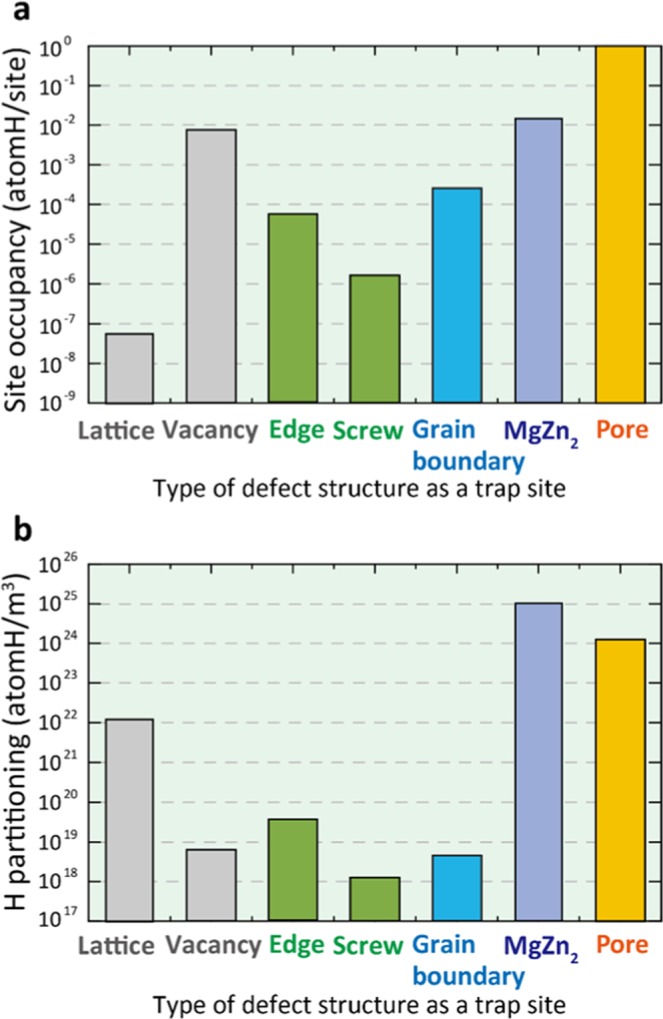
Hydrogen partitioning behaviour in Al-Zn-Mg alloys. Theoretically-derived estimates of (**a**) H occupancy and (**b**) hydrogen partitioning at various defect sites including all contributions of the binding energy shown in Fig. 2(b) and the trap site density for various sites. Site occupancy and actual concentration were derived based on thermodynamic equilibrium condition and first-principles calculations.

As it is still difficult to examine experimentally how hydrogen atoms are actually distribute in the interior area of materials, we implemented the thermodynamic approach incorporated with atomistic simulations. The HE behaviour is dominated by hydrogen partitioning. The equilibrium states of hydrogen partitioning can be expressed as ${C}_{{\rm{tot}}}={\theta }_{L}{N}_{L}+\sum {\theta }_{i}{N}_{i}+{C}_{{\rm{pore}}}$, where *C*_tot_ is the total hydrogen content in an Al alloy, and *θ* and *N* represent the occupancy and the trap site density for the interstitial site *L* and the *i*th trap site, respectively. We evaluated the binding energies of hydrogen at various trap sites using DFT calculations, and evaluated the site occupancy directly using the expression for equilibrium segregation: ${\theta }_{i}/(1-{\theta }_{i})={\theta }_{L}\exp ({E}_{i}/RT)$, where *R* is the molar gas constant, *T* is the temperature, and *E*_*i*_ corresponds to the binding energy of various trap sites evaluated by DFT calculations in Fig. 2(b). The theoretical estimate of site occupancy is given in Fig. 3(a). Site occupancy differs widely, reflecting the exponential contribution of the binding energies.

Next, we estimated the actual trap site densities directly by means of experimental observation. The area of the grain boundary, dislocation density, pore morphology, vacancy concentration, and the area of the precipitate interface were evaluated by experimental techniques and mathematical models (see Supplementary Fig. S1). Total grain boundary area per unit aluminium volume was calculated based on grain size. The dislocation densities were evaluated using the Williamson-Hall method^35^. The detailed procedure to estimate dislocation density was explained in our previous study^36^ and the value was estimated to be 4.07 × 10^14^ m^−2^ ^31^. The volume and surface area of all the pores present in the gauge region of the prepared alloys were analysed using the marching cubes algorithm^37^, where the three-dimensional morphology of pores can be measured by synchrotron X-ray tomography. The area of the η-MgZn_2_ precipitate interface was calculated from TEM observations^29^. The diameter and height of the η-MgZn_2_ precipitate were observed to be approximately 20 nm and 5 nm, respectively.

Note that the trap site density needs to be measured for each sample since it differs depending on the conditions of both the deformation processing and heat treatment. Accordingly, we can estimate hydrogen partitioning at various defect sites by taking all contributions of the binding energy and trap site density for various sites, as shown in Fig. 3(b), where each trapped hydrogen content, *C*_*i*,_ is given by *θ*_*i*_*N*_*i*_. Our estimation indicates that hydrogen atoms are trapped prominently at the Al–MgZn_2_ interface due to the high presence of MgZn_2_ precipitates, while the occupancy of the pore region itself is highest according to the result of the binding energy. Surprisingly, hydrogen atoms tend to be trapped at the interface more than at the grain boundary. The interfacial cohesive energy of the coherent Al–MgZn_2_ interface is sufficiently high but slightly smaller than that of the grain boundaries (see Supplementary Information); therefore hydrogen partitioning has a dominant influence on both intergranular and quasi-cleavage factures through HE.

### Spontaneous hydrogen-accumulated cleavage

**Figure 4:**
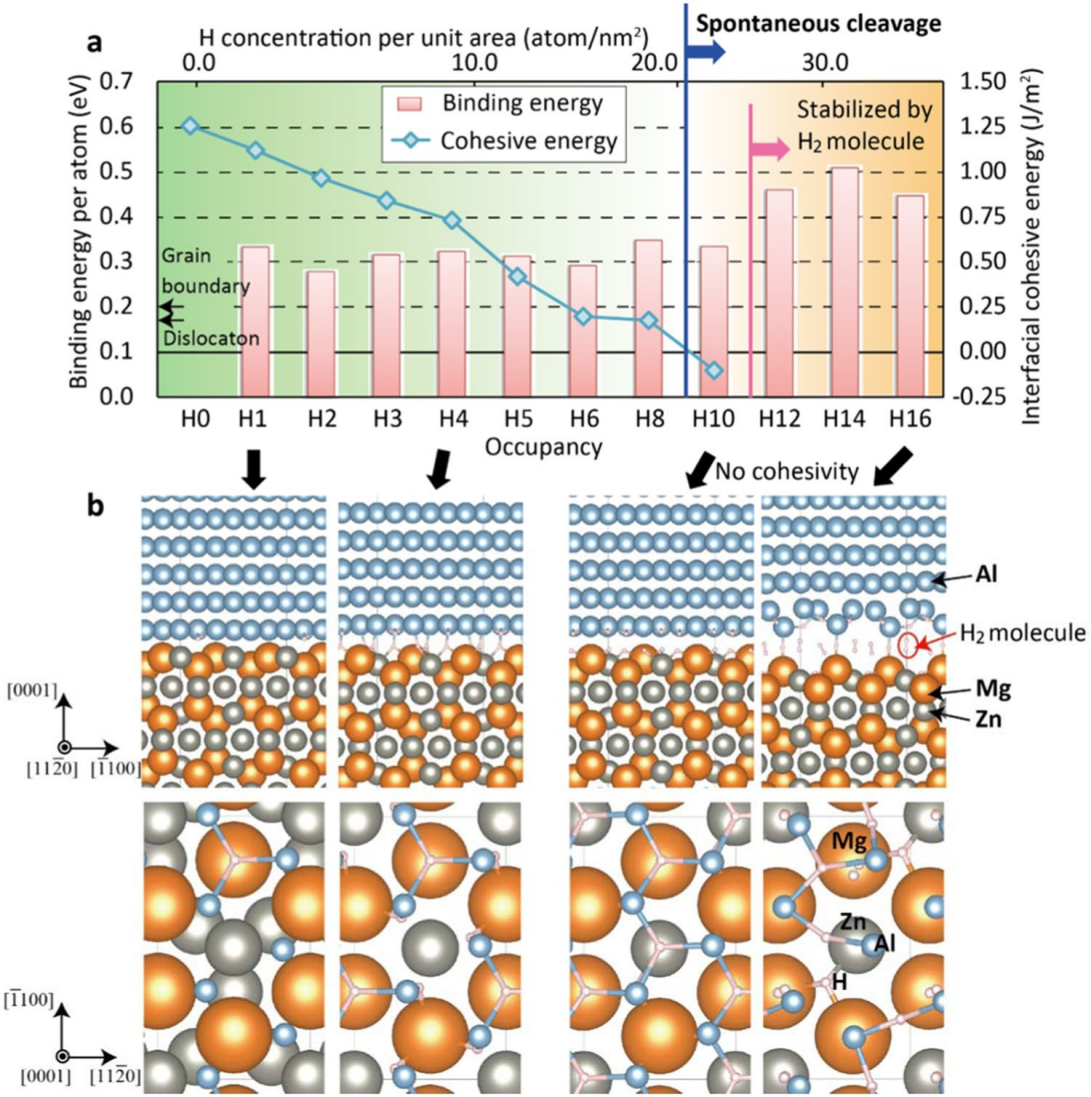
Mechanism of spontaneous cleavage induced by hydrogen segregation at the Al–MgZn_2_ interface. (**a**) Binding energy per atom and interfacial cohesive energy as a function of occupancy. (**b**) The most stable configurations of hydrogen at the interface associated with specific occupancies. Spontaneous cleavage occurs when the concentration of hydrogen atom reaches 22.7 H atoms/nm^2^ according to negative value of the cohesive energy in (**a**). Additionally, the cleavage surface becomes more stable by forming H_2_ molecules at the fracture surface while maintaining hydrogen segregation and spontaneous cleavage.

DFT calculations were again carried out to explore the relationship between cohesive energy and occupancy at the interface and confirm whether such highly-concentrated hydrogen is actually possible at the the Al–MgZn_2_ interface. The binding energy per atom in terms of occupancy is shown in Fig. 4(a). Stable configurations of hydrogen at the interface associated with specific occupancies are given in Fig. 4(b). Despite the case of a coherent interface, the binding energy does not decrease even if the occupancy increases up to the maximum occupancy (ten hydrogen atoms on the interface of the present unit cell). The trap efficiency is still higher than that of other defect structures, such as grain boundaries. In the case that the occupancy exceeds the limit, hydrogen atoms tend to stably nucleate as hydrogen molecules, as shown in Fig. 4(b). As a result, the binding energy continues to increase.

The interfacial cohesive energy corresponding to the occupancy is also given in Fig. 4(a). We found that the cohesive energy decreases as the occupancy increases. These results show that the cohesivity of the interface deteriorates significantly with increasing occupancy, while hydrogen atoms are stably trapped one after the other. As a result, the QCF is induced spontaneously at the Al–MgZn_2_ interface when there is a high concentration of hydrogen atoms. Macroscopic crack propagation was subsequently enhanced as the QCF caused by hydrogen trapping is more active which provides easy crack propagation path related to the direction of the applied stress. This hydrogen-accelerated QCF is unique to the Al-MgZn_2_ interface in Al alloys; it does not occur in Fe alloys because hydrogen is not trapped as significantly at the interface within them^38^. The surface energy of Al is intrinsically lower than that of transition metals^30^ and, moreover, hydrogen is very stable at the surface, which decreases the surface energy. Our findings reveal that hydrogen atoms tend to segregate at the Al–MgZn_2_ interface rather than at grain boundaries and promote hydrogen embrittlement at the interface, resulting in spontaneous microcracking. We believe that to suppress the intensive segregation at the Al–MgZn_2_ leads to design high-toughness Al alloys.

In conclusion, we have discovered a new hydrogen-induced embrittlement mechanism that underpins quasi-cleavage fracture in Al alloys. Despite the coherent atomic configuration, the Al–MgZn_2_ interface is a more preferable hydrogen trap site than other defects in terms of binding energy and hydrogen partitioning. The cohesivity of the interface deteriorates significantly with increasing hydrogen occupancy, while hydrogen atoms are stably trapped up to an extremely high occupancy that is equivalent to spontaneous cleavage. These new findings provide a strategy to design high-toughness Al alloys to suppress HE behaviour in high-strength Al alloys.

## Methods

### Specimen preparation with hydrogen charge

First, Al-10.1Zn-1.2Mg alloys were prepared by homogenization at 773 K for 7.2 ks after casting and hot rolling at 723 K with a rolling reduction of 50%. Alloys after solution treatment were quenched in ice water and immediately subjected to ageing within 10 minutes from quenching. The sample used for the measurement of tomography and EDS maps is prepared at 393 K for 114 ks and 543 K for 25.2 ks aging condition. Specimen preparation and hydrogen charging were performed using electrical discharge machining (EDM, BA8, Mitsubishi Electric, Inc.). The shape of specimens is the same as that reported in the literature^39^, having 0.7 mm in gauge length and 0.6 × 0.6 mm^2^ in cross-sectional area. We confirmed the hydrogen content of the alloy using the vacuum fusion method with EDM, finding that it increased from 0.14 to 6.97 mass ppm in water purified by ion exchange resins^31^. The hydrogen content in the prepared Al-Zn-Mg alloys after EDM cutting by a gas chromatography-type thermal desorption analyser (PDHA-1000, NISSHA FIS, Inc.) was 6.98 mass ppm, which is almost the same as that found elsewhere (6.97 mass ppm)^11^. The hydrogen in specimens after EDM was presumed to be highly concentrated on the surface region. To distribute hydrogen to the interior of the specimens, the specimens were stored in acetone for approximately four days. Considering the diffusion coefficient of hydrogen in aluminium at 300 K^40^, the diffusion distance after four days is estimated to be 2.8 mm, which is enough to diffuse to the inner region of the specimens.

### Microstructural characterization under tensile deformation

The deformation and fracture behaviour of hydrogen-charged Al-10.1Zn-1.2Mg alloys was observed by *in-situ* synchrotron X-ray tomography on BL20XU of Spring-8, Japan. The methodology adopted for tomographic observation and the *in-situ* tensile test follow that reported elsewhere;^11^ a monochromatic 20 keV X-ray was used to perform the tomography. The initial strain rate was set at 3 × 10^−3^ s^−1^ by displacement control, and the test was performed at room temperature. Tomographic observations were taken under several conditions: no loading, near the elastic limit, and every approximately 3% of applied strains until the specimen fractured. The acquired X-ray datasets were three-dimensionally reconstructed by convolutional back-projection^41^. Following tomography, the fracture surface was observed using SEM with 1-kV acceleration voltage. Compositional analysis was performed by EDS at an accelerating voltage of 5 kV in order to observe the precipitates on the quasi-cleavage facets.

### Atomic structure of the Al-MgZn2 interface

The MgZn_2_ precipitate has a C14 Laves phase (space group P63/mmc) in its most stable state^42^. An atomic model of an Al defect-free crystal was constructed with a lattice constant of 4.04 Å. The interstitial site for hydrogen segregation was then chosen as the centre of the tetrahedron in the Al. Coherent interfacial models were subsequently constructed by finding the minimum number of unit cells of both MgZn_2_ and Al that are necessary to minimize the elastic strain along the direction parallel to the interface^34^.

The number of unit cells was ${{\rm{M}}{\rm{g}}{\rm{Z}}{\rm{n}}}_{2}[\bar{1}100]:\,{\rm{A}}{\rm{l}}[1\bar{1}0]=1:\,3$ and ${{\rm{M}}{\rm{g}}{\rm{Z}}{\rm{n}}}_{2}[11\bar{2}0]:\,{\rm{A}}{\rm{l}}[\bar{1}12]=1:\,1$ for η2-MgZn_2_, where the η2 interface could be composed of the smallest multiple numbers of unit cells. The widths of the Al and MgZn_2_ regions along the [0001] and [111] directions were determined so that the length of the multiplied unit cell was larger than 15 Å. We performed preliminary DFT calculations to determine the stable configuration for the other degree of freedom, i.e., the parallel shift on the interface. A vacuum layer with a width of more than 15 Å was inserted, and thus all possible initial configurations were prepared. The Voronoi polyhedral technique was applied to systematically extract possible interstitial sites at the interface using voro++ software^43^.

### DFT calculations for hydrogen trapping

First-principles calculations were conducted within the DFT framework using the Vienna ab initio simulation package (VASP 5.2)^44,45^ with the Perdew–Burke–Ernzerhof generalized gradient approximation exchange-correlation density functional^46^. The Brillouin-zone gamma-centred *k*-point samplings were chosen using the Monkhorst–Pack algorithm^47^, where a 5 × 9 × 1 k-point was chosen for the interface model. A cut-off in plane-wave energy of 400 eV was applied using a first-order Methfessel–Paxton scheme that employed a smearing parameter of 0.1 eV. The total energy was converged within 10^−6^ eV/atom for all calculations. The relaxed configurations were obtained using the conjugate gradient method that terminated the search when the force on all atoms was reduced to 0.01 eV/Å. The zero-point energy of hydrogen atoms was taken into account for the total energy. Atomic configurations were visualized using VESTA 3.4^48^ and Atomeye^49^ software.

## Supplementary information

SUPPLEMENTARY INFORMATION.
