# Supplementary material for: Hydrogen-accelerated spontaneous microcracking in high-strength aluminium alloys
Source: Sci Rep. 2020 Apr 6;10:1998. doi: 10.1038/s41598-020-58834-6 (PMC7136220; doi:10.1038/s41598-020-58834-6)
Supplement: Supplementary file 1 — SUPPLEMENTARY INFORMATION. [file 41598_2020_58834_MOESM1_ESM.docx]

**Supplementary Information**

**Hydrogen-accelerated spontaneous microcracking in high-strength aluminium alloys**

Tomohito Tsuru^1,2,3*^, Kazuyuki Shimizu^4^, Masatake Yamaguchi^5,2^, Mitsuhiro Itakura^5^, Kenichi Ebihara^5^, Artenis Bendo^6^, Kenji Matsuda^6^ & Hiroyuki Toda^4^

^1^ Nuclear Science and Engineering Center, Japan Atomic Energy Agency, Tokai-mura, Ibaraki 319-1195, Japan.

^2^ Elements Strategy Initiative for Structural Materials, Kyoto University, Sakyo-ku, Kyoto 606-8501, Japan.

^3^ PRESTO, Japan Science and Technology Agency, Kawaguchi, Saitama 332-0012, Japan.

^4^ Department of Mechanical Engineering, Kyushu University, Fukuoka, Fukuoka 819-0395, Japan.

^5^ Center for Computational Science and e-Systems, Japan Atomic Energy Agency, Tokai-mura, Ibaraki 319-1195, Japan.

^6^ Graduate School of Science and Engineering for Research, University of Toyama, Toyama, Toyama 930-8555, Japan.

*Corresponding author. e-mail: [tsuru.tomohito@jaea.go.jp](mailto:tsuru.tomohito@jaea.go.jp)

**TEM observation of Al–MgZn2 interface**

The atomic configuration at the Al–MgZn2 interface was investigated by high angle annular dark-field scanning transmission electron microscopy (HAADF-STEM). Alloy Al –3.4 Zn –1.9 Mg (at. %) was prepared by casting in permanent mold. The following processing history was applied: homogenization at 470 °C for 24 h, extrusion at 405 °C, and solution treatment at 475 °C for 1 h followed by quenching into iced-water [1]. The η’ and η2 interfaces were observed under this aging condition. The HAADF-STEM images of η2 interface as a typical case was shown in Fig. S1. The coherent interfaces is obviously observed on (111)_Al_ plane. Coherent interface was also observed at the η’ interface. Similar type of precipitation is expected to be nucleated in the sample used in the main text as the Zn/Mg ratio of the sample is higher than 2 [2]. It was also confirmed that the precipitates can more or less keep its coherency at the interface in wide range of aging condition.


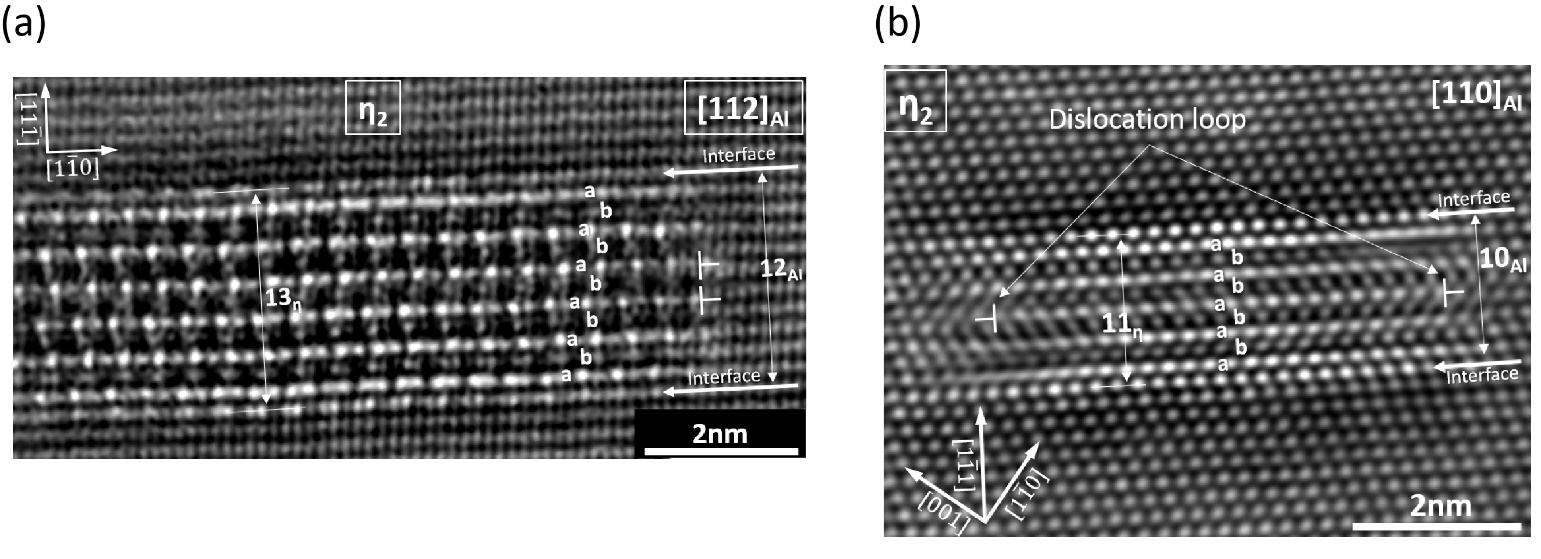


**Supplementary Figure S1** HAADF-STEM **images of** n2 interface in view of (a) [112]_Al_ and [110]_Al_ directions. The completely coherent interface is observed on (111)_Al_ plane.

**Synchrotron X-ray tomography**

Projection-type synchrotron X-ray tomography, illustrated in Fig. S2, was performed using a BL20XU (SPring-8, Japan). The X-ray was passed through a Si (111) double-crystal monochromator, and the energy was tuned to 20 keV. The detector consisted of a 2048×2048 pixel CMOS camera, an optical lens, and a single crystalline scintillator (Lu_2_Al_5_O_12_:Ce). The distance between the specimen and the detector was 20 mm. The spatial resolution of this tomography method is reported elsewhere to be 1.2 μm [3]. A total of 1800 projection images were captured as the specimen was rotated by 180 degrees in steps of 0.1 degrees in the tomographic observation, and these images were reconstructed into a three-dimensional image using a filtered convolution back-projection algorithm [4].


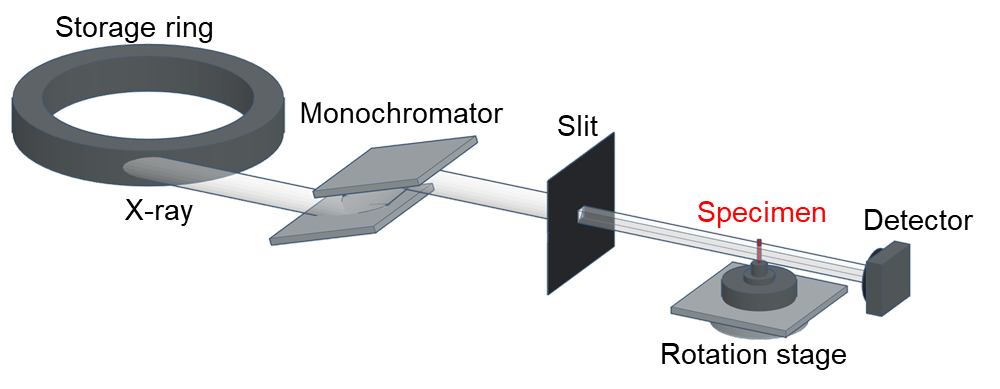


**Supplementary Figure 2** Schematic of the projection-type tomography configuration used. A

monochromatic X-ray is shaped by the slit, transmitted through the specimen on a rotating stage, and detected by the detector.

**Interfacial cohesive energy at interfaces in Al alloys**

Ideal work of separation is the key to predicting the brittle fracture mode associated with intrinsically weak interfaces. The energy-based Griffith criterion [5] for crack propagation was employed, which is derived from the elastic–brittle Griffith model: $G=dU/da\geq dU_{s}/da=2\gamma_{s}$, where *U* and *U*_s_ are the internal energy stored in the elastic media and surface energies. In the case of interfacial fracture, such as a grain boundary (GB), the excess energy of the interface $\gamma_{GB}$ is subtracted from the new fracture surface energy, and the ideal work of interfacial separation $2\gamma_{int}$ can be expressed as $2\gamma_{s}\Rightarrow2\gamma_{s}-\gamma_{GB}\equiv2\gamma_{int}$. DFT calculations can be efficiently applied to estimate the absolute value of the ideal work of fracture, namely, interfacial cohesive energy (2γ_int_).

The embrittlement of interfaces can be understood by considering solution segregation using $2\gamma_{int}$ [5]. The change in surface and interfacial energies corresponds to the segregation energy at the surface and interface, and therefore the hydrogen-induced decohesion can be evaluated directly. Fig. Se shows the interfacial cohesive energies for various interfaces in an Al alloy, including the GB and Al–MgZn_2_ interface. (The surface energies of two separated Al (111) planes are shown for reference.)

It is found that the coherent twin boundary (Σ3(111)) has almost the same cohesive energy as Al (111). The cohesive energy of the MgZn_2_ crystal is also sufficiently high due to its coherent interface, which indicates that the MgZn_2_ crystal and the interface does not cause brittle fracture. On the other hand, it was known that the cohesive energy of the random GB is lower than that of the coincidence site lattice (CSL) GBs. Nevertheless, the quasi-cleavage fracture does not occur at the random GB but even on (111) interface. As a result, the hydrogen partitioning behaviour and hydrogen-induced degradation play dominant roles in the fracture mode of Al alloys.


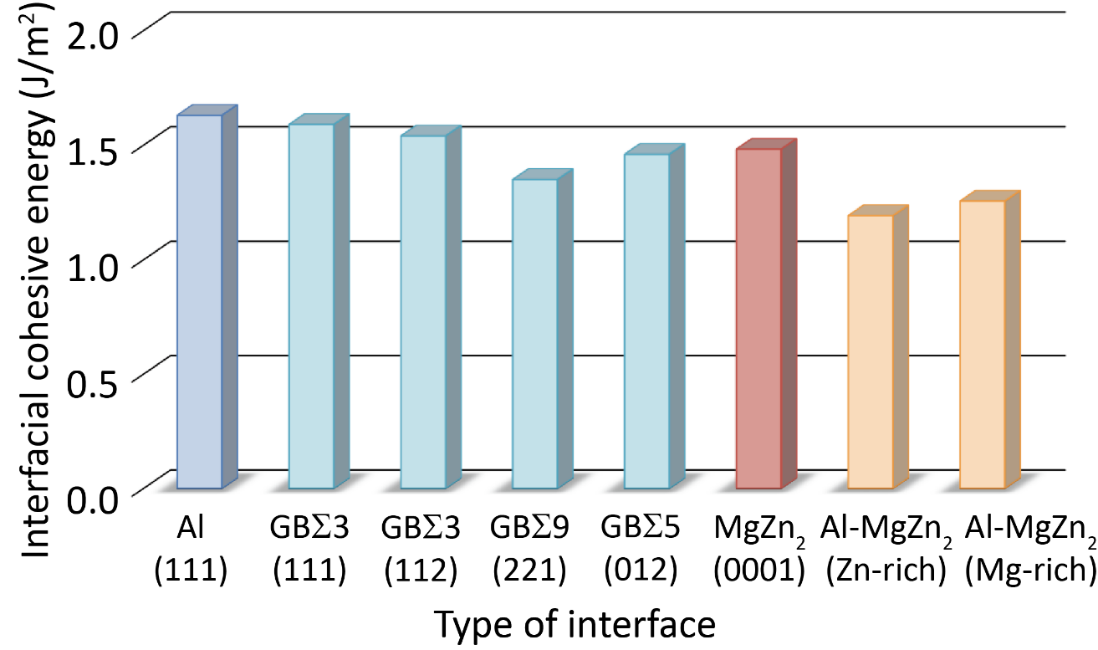


**Supplementary Figure S3** Interfacial cohesive energy at various interfaces in Al alloys without hydrogen. The surface energy for two separated Al (111) planes, various types of GBs, (0001) planes of MgZn_2_ crystals, and Al–MgZn_2_ interfaces were considered as potential fracture surfaces.

**Supplementary References**

1. Bendo, A., Matsuda, K., Lee, S., Nishimura, K., Toda, H., Shimizu, K., et al. Microstructure evolution in a hydrogen charged and aged Al–Zn–Mg alloy. *Materialia* **3**, 50–56 (2018).
2. Berg, L. K., Gjonnes, J., Hansen, V., Li, X. Z., Knutson-Wedel, M., Waterloo, G., Schryvers, D. & Wallenberg, L. R. GP-zones in Al–Zn–Mg alloys and their role in artificial aging, *Acta Mater.* **49** (2001) 3443–3451.
3. Toda, H., Shimizu, K., Uesugi, K., Suzuki, Y. & Kobayashi, M. Application of Dual-Energy K-Edge Subtraction Imaging to Assessment of Heat Treatments in Al-Cu Alloys. *Mater. Trans.* **51**, 2045–2048 (2010).
4. Kak, A.C. & Slaney, M. Principles of Computerized Tomographic Imaging, *Society for Industrial and Applied Mathematics* (2001).
5. Griffith, A.A. The Phenomena of Rupture and Flow in Solids. *Philos. Trans. R. Soc. A* **221**, 582–593 (1921).
6. Rice, J.R. & Wang, J.S. Embrittlement of interfaces by solute segregation. *Mater. Sci. Eng. A* **107**, 23–40 (1989).
